# Secret talk between adipose tissue and central nervous system via secreted factors—an emerging frontier in the neurodegenerative research

**DOI:** 10.1186/s12974-016-0530-x

**Published:** 2016-03-24

**Authors:** Avinash Parimisetty, Anne-Claire Dorsemans, Rana Awada, Palaniyandi Ravanan, Nicolas Diotel, Christian Lefebvre d’Hellencourt

**Affiliations:** Université de La Réunion, UMR 1188, Sainte-Clotilde, F-97490 France; Inserm, UMR 1188 Diabète athérothrombose Thérapies Réunion Océan Indien (DéTROI), plateforme CYROI, Sainte-Clotilde, F-97490 France; Lebanese University, Faculty of Sciences, Beirut, Lebanon; Apoptosis and Cell Death Research Lab, School of Biosciences and Technology, Vellore Institute of Technology University, Vellore, India

**Keywords:** Diabetes, Obesity, White adipose tissue, Adipocytokines, Central nervous system, Neuroinflammation, Neurodegeneration, Neurogenesis

## Abstract

First seen as a storage organ, the white adipose tissue (WAT) is now considered as an endocrine organ. WAT can produce an array of bioactive factors known as adipokines acting at physiological level and playing a vital role in energy metabolism as well as in immune response. The global effect of adipokines in metabolic activities is well established, but their impact on the physiology and the pathophysiology of the central nervous system (CNS) remains poorly defined. Adipokines are not only produced by the WAT but can also be expressed in the CNS where receptors for these factors are present. When produced in periphery and to affect the CNS, these factors may either cross the blood brain barrier (BBB) or modify the BBB physiology by acting on cells forming the BBB. Adipokines could regulate neuroinflammation and oxidative stress which are two major physiological processes involved in neurodegeneration and are associated with many chronic neurodegenerative diseases. In this review, we focus on four important adipokines (leptin, resistin, adiponectin, and TNFα) and one lipokine (lysophosphatidic acid—LPA) associated with autotaxin, its producing enzyme. Their potential effects on neurodegeneration and brain repair (neurogenesis) will be discussed. Understanding and regulating these adipokines could be an interesting lead to novel therapeutic strategy in order to counteract neurodegenerative disorders and/or promote brain repair.

## Background

Obesity and type 2 diabetes mellitus (T2DM) are main health issues in our modern societies and constitute very important public health challenges [1–3]. The World Health Organization (WHO) reported that worldwide obesity has more than doubled since 1980 and more than 1.9 billion adults were overweight in 2014 [2]. One result from excess body weight and physical inactivity is the dramatic development of type 2 diabetes that WHO has predicted to be the seventh leading cause of death in 2030 [3–5]. In parallel, 35.6 million people display dementia and 7.7 million new cases are reported every year, Alzheimer’s disease (AD) being the main cause of dementia [6, 7]. An increasing number of data recently highlights that metabolic syndrome, notably obesity and type 2 diabetes, are correlated with an increased risk to develop dementia and/or neurodegenerative diseases such as AD, as well as neurological and neurovascular disorders [8–11]. Consequently, adiposity has been proposed as an independent factor favoring the development of AD [12–14]. However, breaking the paradigm, recent studies show that underweight people (BMI < 20 kg/m2) display higher risk of dementia while very obese people (BMI > 40 kg/m2) have lower dementia risk than healthy weight people [15]. Similarly, a decrease in BMI from mid-life to late-life has been correlated with an increased risk of dementia [16]. Interestingly, it has been suggested that the misexpression of adipose-derived factors called adipokines or adipocytokines may disrupt directly or indirectly brain homeostasis and functions.

In this review, we aimed at first describing the links between adiposity, adipokines levels, and neurological disorders. Furthermore, adipokine signaling in the central nervous system (CNS), highlighting their potential effects on cognition, neurogenesis, and brain functioning, has also been explored. Finally, the possibilities of adipokines to disturb brain physiology and functions through blood brain barrier disruption resulting from increased inflammation and oxidative stress have been discussed.

## White adipose tissue: not just energy storage

### White adipose tissue secretes adipokines

White adipose tissue (WAT) was originally described to store energy in the form of triglycerides. However, since the discovery of the leptin hormone in 1994, WAT is also recognized as a major endocrine organ secreting a wide variety of biologically active factors collectively called adipokines or adipocytokines [17, 18]. To date, about hundred adipokines constituting the adipokinome have been documented to be released from white adipocytes [19]. The most studied adipokines are leptin, adiponectin, apelin, resistin, monocytes, and macrophage chemotactic protein 1 (MCP1), interleukin-1β (IL-1β), interleukin-6 (IL-6), interleukin-10 (IL-10), tumor necrosis factor-alpha (TNFα), and transforming growth factor (TGFβ). In addition to adipokines, lipid-derived factors (sometimes referred as lipokines) such as the lysophosphatidic acid are also important mediators produced by the fat tissue [20, 21]. Pro-inflammatory factors include adipokines such as leptin, TNFα, and IL-6, while anti-inflammatory ones include adiponectin and the secreted frizzled-related protein 5 (sFRP5) [20, 22, 23]. Adipokines exert pleiotropic effects on different tissues such as the lung, skeletal muscle, heart, liver, and blood vessels and regulate numerous physiological functions such as appetite, energy expenditure, insulin sensitivity and secretion, fat distribution, lipid and glucose metabolism, endothelial function, blood pressure, hemostasis, neuroendocrine functions, and also immunity [18, 21, 24–28]. The data generated over the last 20 years considerably change our view on adipose tissue as WAT plays a wide-ranging role in metabolic regulation and physiological homeostasis [17, 18].

### Adipokines and diseases: focus on neurological disorders and diseases

The dysregulation of adipokine production and/or levels has been correlated with several diseases and could notably promote and/or result in obesity-linked metabolic disorders [27, 29]. Thus, low plasmatic leptin concentrations are associated with an increased risk for cardiovascular diseases [30]. In contrast, higher plasmatic adiponectin levels seem to be associated with decreased risk for developing type 2 diabetes mellitus (T2DM) [31]. Other data also show relationships between MCP-1 serum levels and insulin resistance, as diabetic patients exhibit highest MCP-1 levels [32]. In the same line of evidences, it appears that the inflammatory status of WAT in obese patients might be a key player linking high WAT mass to insulin resistance. Interestingly, an increasing number of studies reported links between metabolic disorders (i.e., T2DM and obesity) and brain homeostasis and functioning [9, 12, 33–39]. Initial studies demonstrated that a higher body mass index (BMI) and/or waist-to-hip ratio in middle-aged individuals is associated with a reduction in the whole brain volume. Indeed, over the last decade, a number of magnetic resonance imaging (MRI) and computed tomography (CT) studies also reported alterations in brain morphology of overweight/obese individuals [40–42]. Studies documented a link between abdominal fat and reduced brain volume in healthy middle-aged adults notably the temporal lobe volume and the hippocampus [43, 44]. In a cross-sectional study of normal elderly individuals showing no sign of cognitive deficit, tensor-based morphometry also unveiled atrophy in the white and gray matter of the frontal lobes, anterior cingulate gyrus, hippocampus, and thalamus in both male and female subjects with a high BMI (BMI > 30) as compared to individuals with a normal BMI (18.5–25) [45]. Upon further investigation, the brain volume reduction in gray and white matter was found to be associated with a common variant of the fat mass and obesity-associated (*FTO*) gene [46]. In addition, a growing body of studies also show that obesity in mid-life is a predictor of mild cognitive impairment with aging and altered executive function and short-term memory compared to normal weight counterparts [39, 47–49]. Such data were also confirmed in rodents for which high fat diets result in impaired cognitive functions including a decrease in memory performance, learning, and executive functions [39, 50, 51]. Furthermore, during the development of obesity in rodent models, it appears that neurochemical changes occurs in the brain altering cognition processes, reward neurocircuitry, and stress responsiveness [52]. Consequently, numerous studies described association between rich diets (sugar and/or fat) and cognitive defects in rodents and humans [39, 52], and it seems that such effects of diets could occur through the disruption of neurovascular function [52–54]. In addition, a linkage has been demonstrated between overweight, neuroinflammation, and neurodegenerative diseases namely AD, Parkinson’s disease (PD), and autoimmune nervous system diseases such as multiple sclerosis [9, 12, 33–38]. Similarly, T2DM is associated to impaired cognition, especially learning and memory deficits such as shown in rodents and humans while such effects are rarely observed in type 1 diabetes [55, 56]. This is peculiarly interesting given that T2DM patients are mostly overweight or obese compared to type 1 diabetic. In a recent study, working on 80 T2DM patients and 80 healthy controls demonstrated a cortical and subcortical atrophy and that cognition impairment was correlated with reduced hippocampal CA1 size in the diabetic group [57]. Diabetes is associated with an increased risk of AD and vascular dementia, supported by increasing oxidative stress and inflammation and impaired insulin and amyloid metabolisms [56, 58–62]. T2DM patients also display lower cerebral blood flow and neural slowing on recordings of sensory-evoked potentials [56]. Numerous studies performed on rodents also show an impact of diabetes on neurogenesis, depression, and cognition [63].

Taken together, these data show that obesity and diabetes have negative effects on brain structures and/or functions. It also raises the question about the roles of adipokines in such neurological disorders. A possible explanation could be that abnormal adipokine concentrations, such as increase pro-inflammatory adipokines TNFα, resistin, leptin, IL-1β, and also IL-6, could influence the blood brain barrier integrity and disrupt brain homeostasis through oxidative stress and inflammation [12, 20]. In the following part, we aim to describe the effects of some adipokines in the brain, regarding their transport in the central nervous system and their signaling.

## Adipokines and targets in the brain

**Fig. 1:**
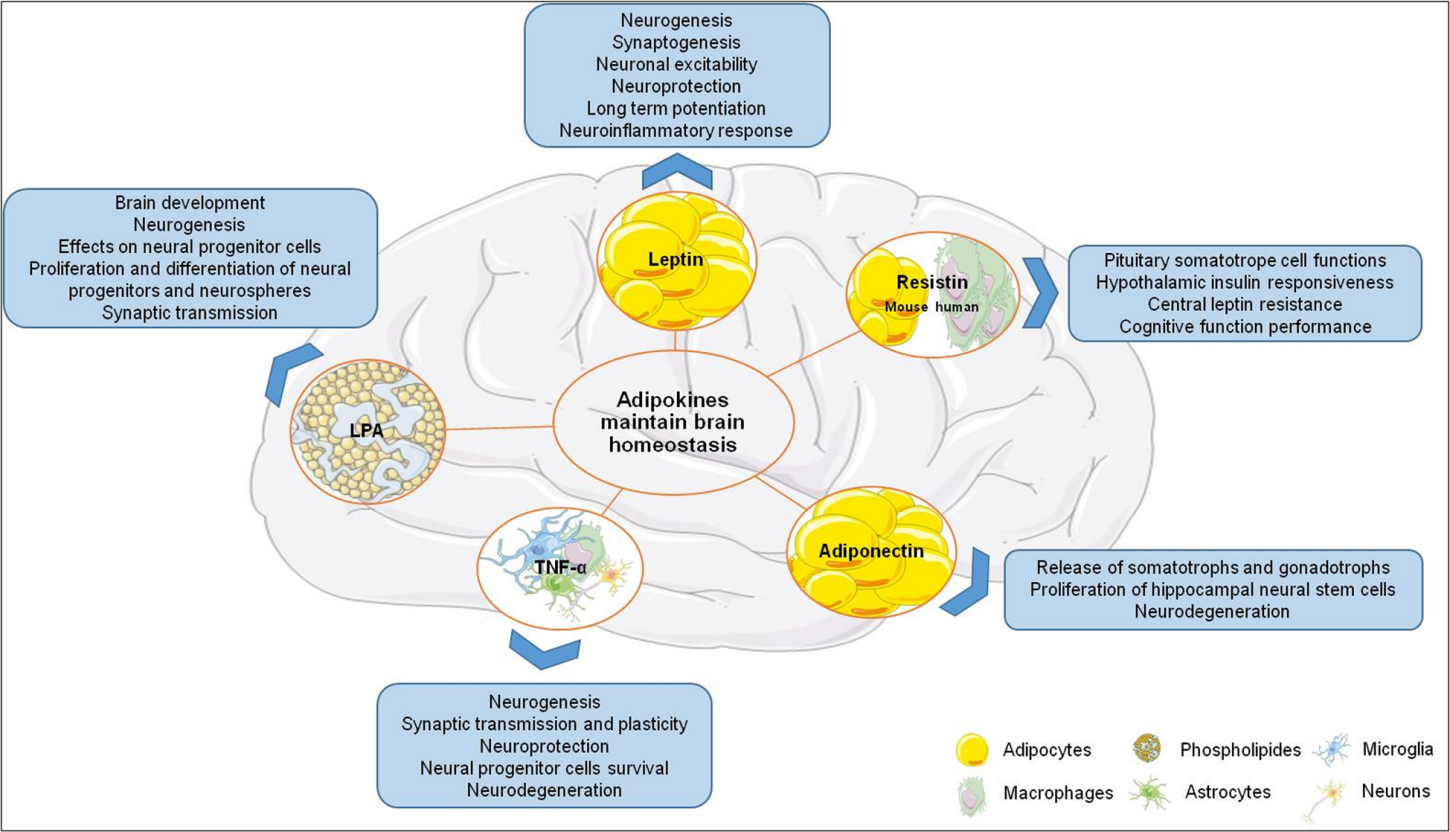
Effects of the main adipokines on brain homeostasis/functions. LPA lisophosphatidic acid, TNFα tumor necrosis factor-α

In this part, we focus on specific adipokines (leptin, resistin, adiponectin, TNFα) and also on a lipokine of interest, the lysophosphatidic acid (LPA) and their targets in the brain as well as their potential impact on brain inflammation and functions (Fig. 1).

### Leptin

Leptin is probably the most studied adipose-derived hormones. Leptin which is mainly produced by adipocytes exerts its effects both peripherally and centrally [17, 21, 24, 64]. This adipokine plays a key role in regulating energy intake and expenditure, metabolism, and behavior by directly acting on the CNS. Mice invalidated for leptin (ob/ob mice) display obesity, insulin resistance, and hyperphagia showing notably the impact of this adipose-derived hormone on feeding behavior [65]. Peripheral leptin exerts its central effect through its binding at the level of choroid plexus leading to its transport across the blood brain barrier [66–69]. Such a transport involved leptin receptors and probably other mechanisms that are still poorly understood [9]. However, some studies have shown that leptin could be also locally and *de novo* produced in the brain, in the cerebellum, the cortex, and the hypothalamus [70–73], suggesting other specific and local functions for leptin than those previously described. Leptin receptors belong to the family of cytokine receptors, and at least five different isoforms have been identified in mouse: Ob-Ra to Ob-Re [65, 74]. In the CNS, leptin receptors (Ob-R or LepR) were first identified in choroid plexus and in the hypothalamus [75, 76]. Among all Ob-R isoforms, only the full-length isoform (Ob-Rb) appears to fully transduce the activation signal at least in the brain and is essential for leptin’s weight-reducing effects [65, 74]. Ob-Rb is expressed in the hypothalamic nuclei notably in the arcuate nucleus (ARC), the dorsomedial nucleus (DMH), the paraventricular nucleus (PVN), the ventromedial hypothalamic nucleus (VMH), and the lateral hypothalamic nucleus (LH) [65, 77, 78] but is also detected in the neocortex, the hippocampus, the hindbrain (nucleus of the solitary tract), the ventral tegmental area, the medulla, and the cerebellum [77, 79–83]. In addition, a weaker expression was also detected by *in situ* hybridization in the hippocampus and the thalamus [77]. The expression of leptin receptors and leptin mRNAs is documented in the mouse brain and notably in the main neurogenic niches, the subventricular zone of the lateral ventricles, and the dentate gyrus of the hippocampus (Allen Brain Atlas [http://www.brain-map.org], [84]). This work clearly illustrates the expression of leptin receptors in the cortex, along the ventricular walls and also in the hippocampus. Leptin is expressed in the same regions at lower levels. In the hypothalamus, the primary leptin targets are the orexigenic agouti-related peptide (AgRP) neurons and the anorexigenic pro-opiomelanocortin (POMC) neurons that are involved in feeding behavior. Thus, in the CNS, leptin activates anorexigenic POMC neurons through a neural network in the arcuate nucleus [85]. The appetite-stimulating effects of AgRP/NPY are inhibited by leptin in the arcuate nucleus avoiding the release of orexigenic factors [86, 87]. Furthermore, leptin receptors were also expressed in glutamatergic and GABAergic neurons [78, 88, 89]. Vong and colleagues (2011) have shown that the main effects of leptin are mediated by GABAergic neurons and only barely by glutamatergic neurons [88]. However, it was recently demonstrated that glutamate release mediates leptin action on energy expenditure [89]. We realize now that the effects of leptin on these different neuronal types and brain nuclei are not so easy to understand as originally thought. In homeostatic conditions, leptin inhibits food intake, and in extra-hypothalamic sites, leptin acts on neurogenesis, synaptogenesis, neuronal excitability, and neuroprotection [9, 90, 91]. Leptin was also shown to improve cognition and mood in depressed and anxious animal models, notably by improving long-term potentiation [9]. Leptin levels negatively correlated with the development of Alzheimer’s disease in lean humans [91, 92], and leptin signaling seems to be dysregulated in Alzheimer’s disease brains [93]. Interestingly, there are also positive correlations between plasma levels of leptin and body weight [94, 95].

### Resistin

Resistin (or adipose tissue-specific secretory factor: ADSF or C/EBP-epsilon-regulated myeloid-specific secreted cysteine-rich protein: XCP1) is a cysteine-rich adipose-derived peptide hormone, encoded by the *RETN* gene, and known for its implication in inflammatory processes [20, 96]. Its expression increases in parallel to adiposity [97–99] and is strongly related to insulin resistance in obese rodents [100]. Interestingly, in humans, resistin is mainly expressed and secreted by macrophages while adipocytes are the main source in rodents [100]. Resistin is known to play a key role in the CNS notably by regulating pituitary somatotrope cell functions [101], affecting hypothalamic and peripheral insulin responsiveness, thermogenesis, and feeding behavior, and also by enhancing renal sympathetic nerve activity [102–104]. However, the resistin receptor and the molecular mechanisms sustaining such effects are poorly understood and mainly unexplored until recently. Although resistin receptor has not been clearly identified, some potential candidate receptors have been proposed in different cell types such as an isoform of decorin (a small proteoglycan associated with collagen fibrils) in adipose progenitor cells, tyrosine kinase-like orphan receptor-1 (ROR1) in 3T3-L1 cells [105] or IGF-1R in fibroblast [106]. Nevertheless, it has been shown that resistin administration modulates or activates several signaling pathway involving Gs protein-dependent mechanisms, the adenylate cyclase/cAMP/protein kinase A pathway, the phosphatidylinositol 3-kinase/Akt pathway, the protein kinase C, and extracellular Ca^2+^ signaling through L-type voltage-sensitive Ca^2+^ [102, 107]. Such puzzling data strongly suggest that resistin could potentially interact with different receptors depending on tissue and cell types. Furthermore, resistin also regulates the synthesis and secretion of the pro-inflammatory cytokines TNFα and IL-6 through nuclear factor-κB-dependent pathway in macrophage [108–110]. Recently, Toll-like receptor 4 (TLR-4) receptors were identified as potential receptor for resistin in the hypothalamus, leading to the activation of JNK and p38/MAPK pathways [111]. Interestingly, resistin was also reported to be expressed in the hypothalamus and the cortex and to inactivate hypothalamic neurons [112–114]. In the rat brain, resistin is *de novo* produced suggesting specific roles for this local synthesis [114]. Resistin gene expression in the brain of mouse is reported in the cortex along the walls of the lateral ventricles and also in the hippocampus (Allen Brain Atlas [http://www.brain-map.org], [84]). In rat, traumatic brain injury (TBI) increased *resistin* mRNA expression in the ipsilateral cortex without any effects on the contralateral hemisphere. However, resistin expression is upregulated after TBI in the ipsi- and contralateral hippocampus [73]. One explanation is that given TBI compromises the integrity of the blood brain barrier, it could result in the changes in gene expression in the contralateral side of the hippocampus by exposing the brain to circulating factors of peripheral origin (Brown et al., 2008). The relatively rapid increase of resistin expression following TBI (at 12 h post-injury), is in contrast to the delayed upregulation of resistin in hypoxic ischemic mouse brain (>7 days) [115]. Thus, resistin could participate in the acute responses to cerebral damage probably through inflammatory mechanisms. A recent study suggested that resistin was not related to cognitive function performance [116].

### Adiponectin

Adiponectin was first characterized in 1995 in 3T3-L1 adipocyte differentiation [117]. It is one of the most abundant adipokines considering its concentration in plasma relative to many other hormones [118, 119]. Adiponectin self-associates into larger structures forming homotrimers that also self-associate and form hexamers or dodecamers. A globular fraction, named globular adiponectin, resulting from the cleavage of the full-length monomer, was also documented [120]. Adiponectin is mainly synthesized and secreted by adipocytes. However, it is now well admitted that adiponectin is expressed at the mRNA and/or protein level by the placenta, the liver, epithelial cells, osteoblasts, myocytes, and also by pituitary cells [114, 119, 121]. Interestingly, some studies documented adiponectin transcript expression in the diencephalon of chicken [114, 122] and in the human pituitary [121]. In the pituitary, adiponectin could have a role in the release of somatotrophs and gonadotrophs [119]. It also modulates a wide range of metabolic processes such as body-weight regulation, glucose regulation, insulin sensitivity, lipid catabolism (fatty acid oxidation), endothelial function, and also anti-atherogenic process [119, 123–126]. Such effects are mediated by three different receptor types: adiponectin receptor 1 (Adipo-R1), adiponectin receptor 2 (Adipo-R2), and T-cadherin (CDH13) and involved different signaling pathways including AMPK, p38-MAPK, JNK, PPAR-α, and NF-kB. These receptors appear to be widely expressed in the mammalian brain including mouse, rat, pork and human. Their expression was documented in different brain structures such as the pituitary, the hypothalamus, and in cortical and subcortical neurons [97, 119, 121, 127–131]. In their review, Thundyil and colleagues (2012) documented adiponectin receptor expression in the central nervous system showing that Adipo-R1 is mainly expressed in the hypothalamus, the brainstem, and the pituitary gland while Adipo-R2 seems to be mostly expressed in the cortex. Furthermore, Adipo-R1 is strongly expressed in neurons and to a lesser extent in astrocytes while Adipo-R2 is figured to be only weakly expressed in astrocytes and neurons [119]. Adiponectin gene expression is widely expressed in the cortex and the hippocampus. Concerning T-cadherin receptor, it seems to be temporally and spatially expressed in different neuronal populations during axon growth [132]. Furthermore, T-cadherin showed broad expression in the cerebral cortex, basal ganglia, amygdala, and hippocampus in the developing postnatal telencephalon of marmoset (*Callithrix jacchus*) [133]. In mouse, CDH13 was also expressed by projection neurons within the main and accessory olfactory bulbs. Interestingly, adiponectin deficiency is associated with exaggerated inflammatory response in critical illness or septic patients [134–136]. Recently, Adipo-R1 and Adipo-R2 expression was described in both U373 MG (human glioblastoma astrocytoma cell line) and primary human astrocytes [137]. It also appears that adiponectin induces a pro-inflammatory response in human astrocytes, increasing notably IL-6 and MCP-1 through NF-κB, p38MAPK, and ERK1/2 pathways (Wan et al., 2014). In contrast, adiponectin was described to inhibit pro-inflammatory signal, notably by suppressing IL-6 release from blood brain barrier (BBB) endothelial cells [138]. It results that adiponectin indirectly modulates inflammatory signaling across the BBB by negatively modulating IL-6 and TNFα release. In vitro experiment of hippocampal neurons reveals that adiponectin exerts neuroprotective effects through AMPK pathway [139]. Such neuroprotective effects of adiponectin are further reinforced by the fact that knock-out mice for adiponectin exhibit more brain damages after ischemic stroke to controls [140]. This neuroprotective action is mediated through an endothelial nitric oxide synthase (eNOS)-dependent mechanism [140].

### Tumor necrosis factor α

Many pro-inflammatory factors are produced in activated WAT, such as TNFα, IL-1, and PGE2. We choose to describe in more depth the prototype inflammatory cytokine TNFα. TNFα is a pro-inflammatory adipokine well-known for its role in chronic peripheral and central inflammation [9, 141]. TNFα is primarily produced as a transmembrane protein that self-associated into stable homotrimers [142, 143]. Such homotrimers could be cleaved by the TNFα-converting enzyme (TACE, also called ADAM17), allowing the release of secreted form of TNFα [144]. In WAT, TNFα is produced by macrophages as well as by adipocytes, and its expression is increased at the mRNA and protein levels in obese and in T2DM models [145]. TNFα actions are mediated by two receptors: TNF-R1 (TNF-RSF1a) and TNF-R2 (TNF-RSF1b). TNF-R1 is expressed in most tissues and can be fully activated by both the membrane-bound and soluble trimeric forms of TNF, while TNF-R2 is found in a limited cell types including cell of the immune system, oligodendrocytes, and certain neuron subtypes and responds to the membrane-bound form of the TNF homotrimer [9]. TNF-R1 and TNF-R2 are also expressed in the cortex, the subventricular zone of the lateral ventricle, and the hippocampus (Allen Brain Atlas [http://www.brain-map.org], [84]). In homeostatic conditions, the TNFα gene expression is low. However, in stress conditions (infection, trauma, pathologies), TNFα level can increase dramatically. As most information regarding TNF signaling is derived from TNF-R1, the role of TNF-R2 is likely underestimated. In rodents, TNFα has been shown to be transported across the BBB, but to be also locally produced by microglia, astrocytes and neurons in the brain [146–148]. In the CNS, TNFα acts through TNF-receptors on neurons and astrocytes regulating a wide range of cellular processes such as cell survival [9, 149, 150]. Actually, TNFα exhibits pleiotropic effects with positive and negative outcomes on the brain. On the one hand, TNFα is considered as the prototypic inflammatory cytokine and elevated levels of TNF have been described in many neurodegenerative situations [151, 152]. For instance, we have demonstrated the role of TNF in chemically induced neurodegeneration [153]. On the other hand, inhibition of TNF in inflammatory peripheral diseases induced CNS side effects including demyelination and neuropathies, suggesting a positive role for TNF maintaining the homeostasis in the CNS [154]. It acts on neurogenesis, synaptic transmission, and plasticity [9]. Thus, TNFα was described for its neuroprotective roles on hippocampal neurons by suppressing the accumulation of reactive oxygen species (ROS) and by maintaining intracellular levels of calcium [155]. In addition, it modulates glutamatergic transmission [156]. Furthermore, TNFα favors neural progenitor cell survival by mediating anti-apoptotic signals via TNF-R2 [157]. In rat, TNFα appears to promote the survival of stroke-generated hippocampal and striatal neurons [158]. In addition, TNFα knock-out mice show cognitive impairment (i.e., significant poorer learning, retention, and spatial learning), suggesting a strong role for TNFα on these mechanisms [159]. However, TNFα also exhibits a dark face, as reported in numerous other studies. It is notably involved in myelin damages [160], in favoring glutamate excitotoxicity [161], in inhibition of long-term potentiation in Cornu Ammonis area 1 (CA1) and in the dentate gyrus of the rat hippocampus [150, 162, 163] and in decreasing neurogenesis [164, 165].

Altogether, these data established that the role of TNFα is complex. TNFα could exhibit multiple faces exerting neuroprotective versus neurotoxic roles, pro- versus anti-neurogenic effects according to the conditions (concentrations, physiological, or pathological conditions…). Neuroinflammation and metabolic disorders such as obesity could act on these mechanisms through an excess of TNFα secretion.

### Lysophosphatidic acid

Among the factors secreted by the adipose tissue, there are many lipids from the lipokine family such as prostaglandin E2 (PGE2), anandamide, and also lysophosphatidic acid (LPA). LPA is a bioactive signaling phospholipid acting on a wide range of biological processes including cell growth, migration, and morphology [166]. LPA is detected in several biological fluids and tissues including the brain [167]. It is synthesized from different enzymatic activities involving notably phospholipase A1 and A2, monoacylglycerol kinase, but the main enzyme leading to LPA synthesis is autotaxin [168]. Autotaxin is a multifunctional phosphodiesterase that converts lysophospholipids into LPA through its lysophospholipase D activity. To date, LPA effects are mediated through five G protein coupled receptors. However, additional receptors have been identified for their potential responsiveness to LPA [168–170]. Using knock-out mice for the five most known LPA receptors (LPA-R), it was shown that LPA plays key roles on inflammation [171], angiogenesis [172], reproduction [173–175], brain development, and neurogenesis [176, 177]. Indeed, LPA exerts pleomorphic effects on neural progenitor cells from cortex, and notably calcium-mediated conductance [178]. In the nervous system, neural progenitor cells, neurons, oligodendrocytes, Schwann cells, astrocytes, and microglia have been documented for expressing different subsets of LPA receptors [168]. It partially explains why LPA exerts a wide variety of effects on these different cell types. Thus, LPA can favor proliferation and differentiation of neural progenitor cells as shown by treatment on *ex vivo* embryonic brain slice cultures resulting in an increase cell survival and differentiation [179]. Furthermore, LPA has been shown to promote proliferation and differentiation in neurospheres [180, 181]. LPA also displays effect on cell morphology and neurite formation in both neural progenitor cells and neurons [168]. It exhibits both cell death and survival properties on neurons possibly due to differences in LPA concentration or signaling through different receptors [182–184]. For instance, it induces apoptosis and necrosis in hippocampal neurons [182]. LPA also exerts various effects on glial and microglial cells, by modulating intracellular calcium levels in oligodendrocytes, astrocytes, and microglia [168]. It notably favors astrocytes and microglia proliferation in vitro [185, 186]. Overexpression of autotaxin in microglia and by consequences, increased levels of LPA, protects the cells from an oxidative stress by increasing the level of catalase [187] and decreases the inflammatory response at least partially through an upregulation of IL-10 [188]. Interestingly, following brain injury, in human postmortem brains, LPA receptors 1–3 and autotaxin are only weakly expressed while LPA-R2 is increased and autotaxin transcripts are decreased. Such data also reinforce the fact that LPA signaling is involved in neurotrauma [189]. During embryogenesis, LPA-R1 was detected in neural progenitors reinforcing a potential role of LPA/LPA-R1 signaling in neurogenesis [190]. In addition, LPAR-1 knock-out reduces brain cell proliferation, differentiation, and cell survival in the mouse dentate gyrus, consequently strongly impairing neurogenesis [176]. Autotaxin is widely expressed in the brain of mouse notably in neurogenic niches while LPA-R1 displays a lower and more discrete expression (Allen Brain Atlas [http://www.brain-map.org], [84]).

## Blood brain barrier, adipokines, inflammation, and oxidative stress

**Fig. 2:**
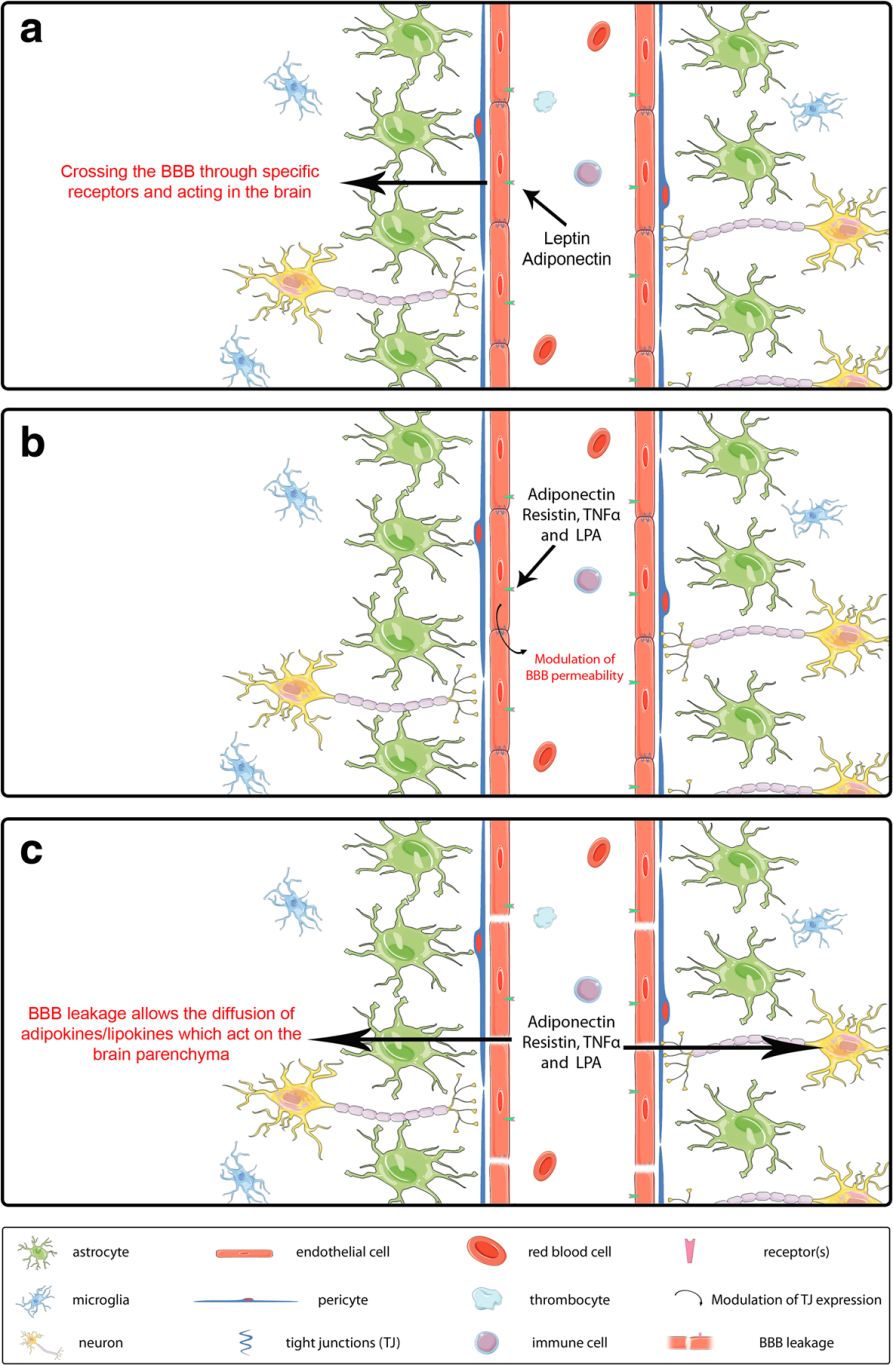
Adipokines and LPA interactions with the blood brain barrier and the central nervous system. The BBB is composed of endothelial cells (displaying tight junctions), pericytes and astrocytes. **a** In physiological conditions, some adipokines such as leptin and TNFα can cross the BBB through different mechanisms and act on the central nervous system. **b** Adipokines can also activate endothelial cell receptors resulting in the modulation of the expression of tight junctions and in the modulation of the BBB permeability. In adiponectin case, one study reports its crossing through the BBB [191], while another indicates that it does not cross the BBB [138]. Both possibilities are shown. In inflammatory conditions, the BBB is leaking and could allow an increase passage of adipokines and LPA into the CNS, leading to an increase of oxidative stress and neurodegeneration (**c**)

The effects and actions of adipokines on the CNS are dependent on their capacity to interact with the cells of the BBB and eventually to enter the CNS (Fig. 2). As mentioned above about the TNFα, some adipokines including leptin can cross the BBB [9]. Concerning adiponectin, its capacity to cross the BBB is questionable. In one study, the results based on the measurement of radioactive labeled adiponectin and concentration in the cerebrospinal fluid indicate that adiponectin does not cross the BBB but will affect the cells of the BBB and indirectly affect the CNS acting via AMPK pathway [9, 138]. In another work using adiponectin KO mice and recombinant adiponectin, it is shown that adiponectin crosses the BBB [191]. As the conclusion, these two publications proposed opposite results concerning the possibility of adiponectin to cross the BBB, and more conclusive results will be needed to clarify this point.

The BBB is a key player in the adipokine signaling from the periphery to the CNS. Inflammation and oxidative stress associated with pathologies impact neurodegeneration and neurogenesis but also affect the BBB.

In this review, we highlighted the striking correlations between metabolic syndrome and the prevalence of neurological disorders and dementia including AD. White adipose tissue was not initially envisioned as a source of inflammatory factors. However, it is now well accepted that WAT is a key player in the development of a chronic low-grade inflammation associated with adiposity and consequently obesity [192, 193] with elevated production of pro-inflammatory cytokines, such as TNFα, IL-6, and IL-1 [194, 195]. In contrast, loss of WAT is associated with a decrease in inflammation markers [196, 197]. Interestingly, chronic and low-grade inflammation has been proposed to negatively favor neurodegenerative diseases through the disruption of the BBB. Indeed, the blood brain barrier is a key interface linking systemic inflammation, neuroinflammation, and neurodegeneration [198], inflammatory factors being a main cause of the BBB disruption [199]. For instance, studies established positive correlations between mid-life adiposity in women with disruption of BBB integrity, showing that overweight/obesity could favor the onset of vascular disorders increasing BBB permeability later in life [200]. In the same line of evidence, rats fed with Western diet, known for promoting diabetes and obesity, display a leakier BBB due to the decreased expression of tight junctions [201]. Kanoski and colleagues have also shown that a primary cerebral target following BBB disruption is the hippocampus, well-known for its involvement in cognitive processes [201]. This is of peculiar interest given that AD patients display hippocampal atrophy and disruption of fronto-hippocampal connections early in the course of the disease [202–204]. This is further reinforced by the fact that AD in human and rodent models is strongly linked to an increased permeability of the BBB [205, 206]. Consequently, the chronic low-grade inflammation that takes place in obese and diabetic people could negatively favor brain inflammation and degeneration through BBB disruption.

Some interesting links exist between dietary factors displaying anti-inflammatory properties, inflammation, and disease outcomes. For instance, polyphenols such as flavonoids and curcumin and spices such as cinnamon have been suggested to decrease pro-inflammatory cytokines, inflammation, and cardiovascular diseases and type 2 diabetes which are known to be risk factors for AD (for a recent review on this topic see ref [207]).

## Conclusions

While the causal nature of all the processes leading to neurodegeneration has not been definitively established, it is widely accepted that neuroinflammation and oxidative stress responses occur with clinical manifestation of the disease. In this review, we described the impact of pro-inflammatory adipokines (TNFα and leptin) on brain homeostasis and functions. In addition, pro-inflammatory adipokines play a major role in the production of reactive oxygen species (ROS) [208, 209]. Due to its ability to secrete adipokines that promote ROS production, WAT has been regarded as an independent factor provoking oxidative stress [210–212]. Exposure to obesity for a long time in a host system downregulates and depletes the activity of antioxidant enzymes such as superoxide dismutase (SOD), catalase (CAT), and glutathione peroxidase (GPx); these enzymes being found to be significantly lowered compared with healthy persons which in turn lead to the development of obesity-related health problems [213]. In addition to this, levels of vitamin A and levels of serum antioxidants, such as vitamin E, vitamin C, and β-carotene, as well as glutathione, are also decreased in obesity [214]. When compared to the normal or lean individuals, obese individuals exhibit high levels of biomarkers of oxidative damage and inflammation such as C-reactive protein, LDL oxidation, and triglyceride levels [215]. Thus, apart from inflammation, which is quite well-known to be one of the critical factors that damages the brain, ROS production which exceeds the antioxidant defenses in the host system is another factor that can also result in brain damages [216]. Cytokines produced by the monocytes and macrophages in WAT are the potent stimulators for the production of reactive oxygen (ROS) and nitrogen species (RNS) which generates oxidative stress. Adipose tissue also has the secretory capacity of angiotensin II, which stimulates nicotinamide adenine dinucleotide phosphate (NADPH) oxidase activity. NADPH oxidase comprises the major route for ROS production in adipocytes [217]). Thus, obesity results in an increased oxidative stress status that can lead to neural dysfunction and death [218, 219]. It has been reported that obesity may induce systemic oxidative stress and, in turn, oxidative stress is associated with an irregular production of adipokines, which contributes to the development of the metabolic syndrome [220]. In parallel, oxidative stress is implicated in numerous neurological diseases and/or disorders such as AD, PD, amyotrophic lateral sclerosis (ALS), multiple sclerosis (MS), cerebral ischemia/reperfusion injury, and TBI, promoting neurodegeneration [221]. An increasing number of studies using in vitro models and knock-out animals demonstrate that oxidative stress disrupts the BBB permeability [221–223].

Taken together, these data suggest that in pathological conditions, adipokines released by WAT promote inflammation and ROS production that may disrupt the BBB permeability and could directly or indirectly act on different brain structures, the hippocampus being one of the most sensitive areas. It could explain why metabolic syndrome is associated with hippocampus atrophy and an increase risk to develop dementia such as AD. One main issue in people suffering from metabolic syndrome should be to struggle against inflammation and reduce oxidative stress in order to decrease their potential effects on brain neurodegeneration and their adverse effects.
